# Predictive value of preoperative peripheral blood neutrophil/lymphocyte ratio for lymph node metastasis in patients of resectable pancreatic neuroendocrine tumors: a nomogram-based study

**DOI:** 10.1186/s12957-017-1169-5

**Published:** 2017-05-30

**Authors:** Zhou Tong, Lulu Liu, Yi Zheng, Weiqin Jiang, Peng Zhao, Weijia Fang, Weilin Wang

**Affiliations:** 1 Cancer Biotherapy Center, First Affiliated Hospital, School of Medicine, Zhejiang University, Hangzhou, China; 2 Division of Hepatobiliary and Pancreatic Surgery, Department of Surgery, First Affiliated Hospital, Zhejiang University, Hangzhou, China; 3 Collaborative Innovation Center for Diagnosis and Treatment of Infectious Diseases, Hangzhou, China; 4 Key Laboratory of Precision Diagnosis and Treatment for Hepatobiliary and Pancreatic Tumor of Zhejiang Province, Hangzhou, 310003 China

**Keywords:** Pancreatic neuroendocrine tumors, Neutrophil-to-lymphocyte ratio, Lymph node metastasis, Recurrence-free survival

## Abstract

**Background:**

Neutrophil-to-lymphocyte ratio (NLR) is one of the systemic inflammation markers, which has prognostic values in many types of tumor. However, hardly any research has reported the relationship between NLR and pancreatic neuroendocrine tumors (PanNETs). In this study, we aimed to evaluate the predictive value of the preoperative peripheral blood NLR on the clinical outcomes in patients of resectable PanNETs.

**Methods:**

Ninety-five cases of PanNETs registered in the First Affiliated Hospital of Zhejiang University between March 2009 and May 2016 and underwent pancreatic surgery were included in this study. Univariate and multivariate analyses were applied to identify the prognostic factors for PanNETs. Prognostic nomogram and its calibration curve then used R (version 3.3.2) to predict lymph node (LN) metastasis.

**Results:**

Among these 95 patients, 52 (54.7%) patients were diagnosed as grade 1 (G1) NET (mitotic count <2/10 HPF, Ki-67 ≤2%), 32 (33.7%) as G2 NET (mitotic count 2–20/10 HPF, Ki-67 3–20%), and 11 (11.6%) as G3 NEC (mitotic count >20/10 HPF, Ki-67 >20%). Increased NLR was found to relate with advanced T stage, LN metastasis, tumor thrombus formation, and advanced grade (*p* < 0.05 for all). Multivariate logistic regression was performed and indicated that NLR (HR 6.74; *p* = 0.02) was an independent prognostic factor for LN metastasis. Furthermore, we developed a nomogram based on the combination of NLR, T stage, and grade for LN metastasis with a good discrimination ability with the AUC (area under the curve) of 0.885. This nomogram showed larger AUC than those using NLR (0.725), T stage (0.808), or grade (0.708) alone as a prognostic factor, which means this system achieved a more optional performance in predicting clinical outcomes. Finally, the Kaplan–Meier curve indicated that the recurrence-free survival (RFS) of patients with high NLR (NLR >1.40, RFS 61.1 ± 4.4 months) decreased significantly as compared with those of low NLR (NLR ≤1.40, RFS 63.8 ± 2.9 month, *p* < 0.05).

**Conclusions:**

The preoperative NLR is a potential independent predictor for LN metastasis and RFS. Our nomogram highlighted the important role of NLR in prognosis, which might be considered as a convenient indicator for lymph node metastasis, especially during the initial diagnosis for resectable PanNETs.

## Background

Pancreatic neuroendocrine tumors (PanNETs) are rare tumors with distinct clinical syndromes, which may obtain malignant potential [1, 2]. The worldwide incidence and prevalence of PanNETs increased recently, which may have resulted from the improved medical technology of detection [3, 4]. A minority of PanNETs are functional and accompanied with clinical syndrome of hormone excess such as insulin, gastrin, glucagon, somatostatin, or vasoactive intestinal peptide. Meanwhile, the majority of PanNETs are non-functional and asymptomatic as they do not secrete these active compounds. Due to this heterogeneous nature of PanNETs, identifying reliable prognostic features have been a challenge.

Recent studies have demonstrated that the survival and prognostic outcomes are associated with lymph node (LN) metastasis in PanNETs. Many studies showed survival benefits of patients without LN metastasis [5–11]. Moreover, awareness of LN status preoperatively may improve preoperative risk stratification and therefore help to make a treatment decision, especially surgical options between the standard resection with LN harvest (pancreaticoduodenectomy and distal pancreatectomy) versus the parenchyma-sparing resection (enucleation and middle pancreatectomy) [9]. In combination with the two reasons stated above, earlier ascertainment of LN metastasis may attain clinical significance, providing more accurate and promising treatment strategies as early as the diagnosis, as well as obtaining prognostic information. Imaging techniques, as the most popular forms to detect LN metastasis traditionally, which include enhanced computed tomography (CT) and magnetic resonance imaging (MRI), can sometimes only offer limited information on LN metastasis. Their limitations include, but are not limited to, the inconsistent sensitivities and specificities, as well as patients’ potential allergy to the contrast and increased cost [12]. Therefore, proper and convenient predictive markers are needed to evaluate the risk of LN metastasis and survival of PanNETs at the initial time frame of the diagnosis.

The systemic inflammatory response (SIR), another critical factor to predict tumor invasion, metastasis and angiogenesis, has been demonstrated to be closely related with poor outcomes in most cancers over the past decades [6]. The white blood cell population changes such as increased neutrophils and deceased lymphocytes have been well recognized as signs of systemic inflammatory conditions [13, 14]. Because the neutrophil-to-lymphocyte ratio (NLR) can be easily measured, it has been thought as a marker of SIR and therefore intensively investigated in predicting prognostic outcomes and refining the risk stratification for cancer patients before treatment. Elevated NLR has been found to correlate with advanced stage and poor prognosis in many types of solid tumors, such as breast cancer, gastric cancer, pancreatic cancer, prostate cancer, and renal cancer [7, 15–24]. Even though, the association between NLR and the LN metastasis remains to be revealed in PanNETs. Here, we performed this retrospective study to evaluate the predictive value of the preoperative blood NLR on the LN metastatic status and, eventually, to provide evidence for prognosis of resectable PanNETs.

## Methods

### Patients

We retrospectively reviewed 95 cases of resectable PanNETs registered between March 2009 and May 2016 and underwent pancreatic surgery in the First Affiliated Hospital of Zhejiang University. The study followed the international and national regulations in accordance with the Declaration of Helsinki and was approved by the ethics committee of the First Affiliated Hospital, Zhejiang University. Patients’ characteristics were obtained including patient demographics, pathologic TNM staging, histology stage, tumor thrombus, functioning status, blood loss volume, operation duration, surgical approaches, and tumor markers. TNM staging was adopted according to the ENETS Consensus Guidelines [25]. Grade was adopted according to the new WHO 2010 grading classifications [26]. Peripheral blood samples for all 95 patients were collected right after the diagnosis before operation and any other therapeutic methods. We defined normal values of CEA (carcinoembryonic antigen), AFP (alpha-fetoprotein), CA199 (carbohydrate antigen 199), and CA125 (carbohydrate antigen 125) as 0–5 ng/ml, 0–20 ng/ml, 0–37 U/ml, and 0–35 U/ml, respectively.

### Postoperative follow-up

Patients were followed by outpatient clinics or phone calls until September 2016. These follow-ups were achieved with 3-month intervals for the first 2 years and 6-month intervals for 2 to 5 years after operation. Recurrence-free survival (RFS) was defined as the number of months from the date of surgery to the date of identification of disease recurrence (evaluated in outpatient clinics by radiological method: CT or MRI) or the date of last follow-up for progress-free patients. If the patient did not come to clinics at the predicted follow-up time, phone calls were made to ask if the patient had some clinical symptoms and to make sure of the survival status. In this study, the median follow-up duration was 31 (range 3–84) months.

### Data analysis

All statistical analyses and graphics in this study were calculated and demonstrated by using the IBM SPSS Statistics software 20.0 and R software packages (version 3.3.2 URL http://www.r-project.org/). *T* tests were applied to compare variables with a normal distribution, whereas the Mann–Whitney *U* test was utilized to evaluate ranked variables. Univariate and multivariate logistic regression analyses were performed to analyze risk factors for LN metastasis. The Kaplan–Meier method was also adopted to calculate cumulative RFS, and comparisons between groups were achieved with the log-rank test. The ROC curve was used to estimate the performance of NLR to LN metastasis and RFS. On the basis of the results of the multivariable analysis, a nomogram was formulated by R software 3.2.0 with the rms package. The area under the curve (AUC) of the ROC was used to assess the discrimination of the nomogram. The calibration plot with bootstrapping was adapted to illustrate the actual probability and the predicted probability [27]. Differences with two-sided *p* value <0.05 was considered statistically significant.

## Results

### Patient demographics

Of the 95 enrolled patients, there were 39 (41.1%) males and 56 (58.9%) females, with a mean age of 54.4 ± 12.1 years. There were 34, 28, 21, and 12 cases from stages T1 to T4, respectively. Fifteen patients were histologically confirmed to have LN metastasis. There were 52 patients defined as G1 NET (grade 1 neuroendocrine tumor; mitotic count 2/10 HPF, Ki-67 ≤2%), 32 as G2 NET (mitotic count 2–20/10 HPF, Ki-67 3–20%), 11 as G3 NEC (neuroendocrine carcinoma; mitotic count >20/10 HPF, Ki-67 >20%). Six patients were confirmed to have tumor thrombus by H&E stains of the resection specimen. Among the surgical choices for these patients, distal pancreatectomy was the most common surgical procedure (49.5%), followed by pancreaticoduodenectomy, local resection of pancreatic tumor, and total pancreatectomy (28.4, 20.0, and 2.1%, respectively). Radical resection was performed in 89 patients, while non-radical resection (not R0 resection, 6.3%) was performed for six patients. Of these 95 patients, 21 patients (22.1%) had functioning tumors. The operation duration ranged from 77.0 to 752.0 min, with an average of 302.0 ± 158.0 min. Average blood loss volume was 312.8 ± 661.2 ml (Table 1). Since there is no standard adjuvant therapeutic protocol for pancreatic neuroendocrine neoplasms, only eight patients received adjuvant therapy, including chemotherapy and somatostatin analogs. The regimen of chemotherapy was etoposide plus cisplatin or irinotecan.

**Table 1:** Patient characteristics

Characteristics	Patients (cases (%))
Gender	
Male	39 (41.1)
Female	56 (58.9)
Age, years	54.4 ± 12.1
TNM staging of ENETS in 2006	
T1	34 (35.8)
T2	28 (29.5)
T3	21 (22.1)
T4	12 (12.6)
N0	80 (84.2)
N1	15 (15.8)
M0	80 (84.2)
M1	15 (15.8)
Grading classifications of WHO 2010	
NET G1	52 (54.7)
NET G2	32 (33.7)
NEC G3	11 (11.6)
Tumor thrombus	
Yes	6 (6.3)
No	89 (93.7)
Surgical resection	
Radical	89 (93.7)
Nonradical	6 (6.3)
Functioning tumor	
Yes	21 (22.1)
No	74 (77.9)
Blood loss volume, ml	312.8 ± 661.2
Operation duration, min	302.0 ± 158.0
Surgical approaches	
Distal pancreatectomy	47 (49.5)
Local resection of pancreatic tumor	19 (20.0)
Pancreaticoduodenectomy	27 (28.4)
Total pancreatectomy	2 (2.1)

*NET* neuroendocrine tumor, *NEC* neuroendocrine carcinoma

### Preoperative NLR and clinical parameters in PanNET patients

NEC G3 Patients had higher NLR as compared with NET G2 and NET G1 patients (*p* = 0.015). NLR in later T stage (T3 and T4) patients also elevated as compared with earlier T stage patients (T1 and T2) (*p* = 0.044). In addition, NLR elevated in tumor thrombus positive (*p* = 0.002) and LN metastasis (*p* < 0.001) patients as well, as shown in Table 2. As a contrast, the NLR was not significantly associated with tumor markers including AFP and CA125. In summary, higher NLR is related with advanced tumor stage and higher grade.

**Table 2:** Preoperative NLR and clinical parameters

Variables	Number	NLR (mean ± SD)	*p*
Gender			
Male	39	2.165 ± 0.980	0.908
Female	56	2.134 ± 1.474	
Age			
≤60	60	2.102 ± 1.335	0.659
>60	35	2.224 ± 1.218	
TNM staging of ENETS in 2006			
T1, T2	62	1.909 ± 0.843	0.044
T3, T4	33	2.594 ± 1.790	
N0	80	1.912 ± 0.836	<0.001
N1	15	3.399 ± 2.282	
M0	80	2.094 ± 1.290	0.361
M1	15	2.427 ± 1.287	
New grading classifications of WHO in 2010			
NET G1	52	1.862 ± 0.746	0.015
NET G2	32	2.305 ± 1.597	
NEC G3	11	3.031 ± 1.868	
Tumor thrombus			
Yes	6	3.703 ± 1.573	0.002
No	89	2.042 ± 1.207	
Functioning tumor			
Yes	21	2.049 ± 0.874	0.697
No	74	2.174 ± 1.387	
AFP			
Normal	91	2.171 ± 1.308	0.384
Abnormal	4	1.594 ± 0.521	
CA199			
Normal	93	2.163 ± 1.300	0.396
Abnormal	2	1.378 ± 0.251	
CEA			
Normal	91	2.159 ± 1.312	0.665
Abnormal	4	1.872 ± 0.444	
CA125			
Normal	92	2.147 ± 1.300	0.979
Abnormal	3	2.128 ± 1.067	

*NET* neuroendocrine tumor, *NEC* neuroendocrine carcinoma, *AFP* alpha-fetoprotein, *CA199* carbohydrate antigen 199, *CEA* carcinoembryonic antigen, *CA125* carbohydrate antigen 125

### Relationships between LN metastasis and NLR and other clinicopathologic characteristics

**Fig. 1:**
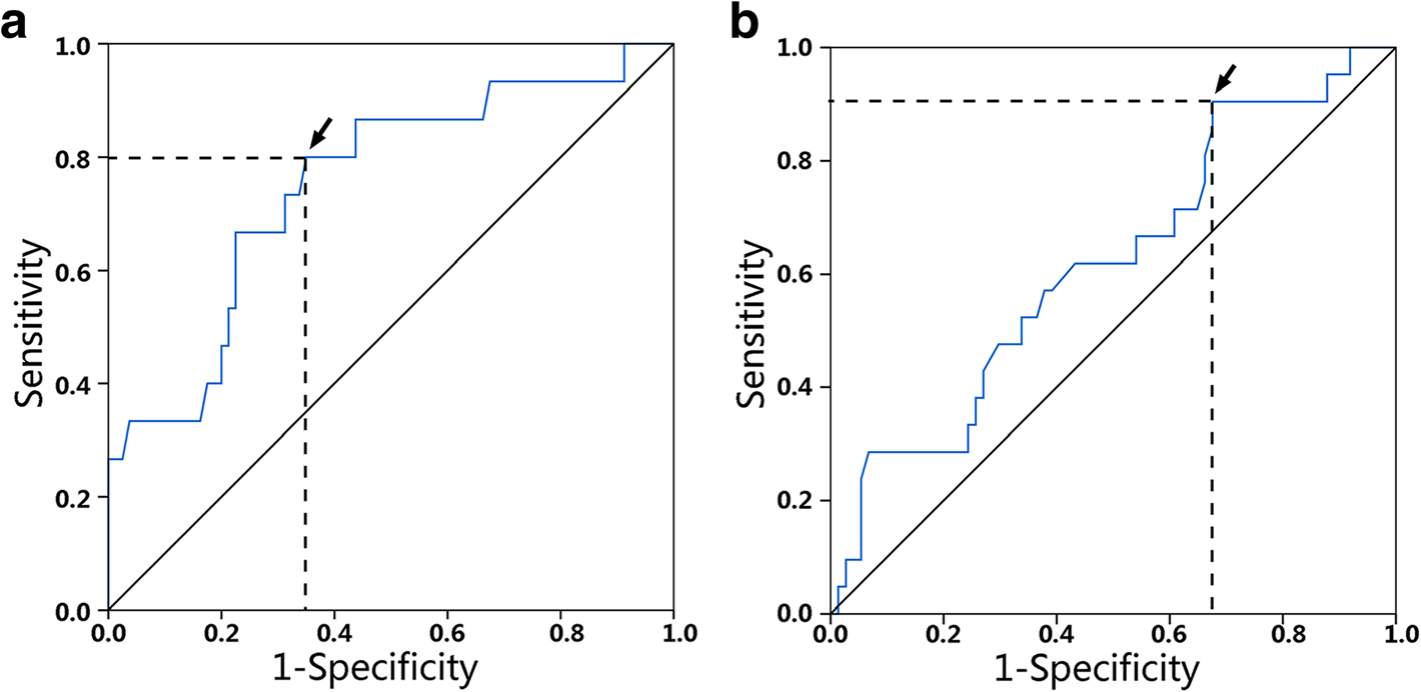
ROC curve for NLR to **a** lymph node metastasis and **b** recurrence-free survival. *Arrows* indicate optimal cutoff values

ROC curve analysis was applied to categorize the optimal cutoff values of NLR for LN metastasis, which was settled as 2.056 (Fig. 1a). Based on this setting, we classified the patients into groups of “high NLR (>2.056)” and “low NLR (≤2.056).” The sensitivity and specificity of the cutoff for NLR were 80 and 65%, respectively. Univariate analysis indicated that NLR (HR 7.429, *p* = 0.003), T stage (HR 19.500, *p* < 0.001), status of tumor thrombus (HR 14.182, *p* = 0.004), or histological grade (HR 16.625, *p* < 0.001) showed significant differences between groups with and without LN metastasis (Table 3). According to the study described above, these four variables were selected as potential independent risk factors in multivariate analysis. Multivariate logistic regression indicated that T stage (HR 11.940, *p* = 0.005) and grade (HR 10.378, *p* = 0.022), as well as NLR (HR 6.740, *p* = 0.023), were all independent prognostic factors for LN metastasis.

**Table 3:** Univariate and multivariate analyses of clinical characteristics according to lymph node metastasis

Characteristics	Univariate		Multivariate	
	HR (CI 95%)	*p*	HR (CI 95%)	*p*
Gender				
Male	2.5 (0.809, 7.722)	0.111		
Female				
Age				
Age ≤60	0.833 (0.260, 2.672)	0.759		
Age >60				
TNM staging of ENETS in 2006				
T1, T2	19.500 (4.047, 93.951)	<0.001	11.940 (2.098, 67.965)	0.005
T3, T4				
New grading classifications of WHO in 2010				
NET G1 + G2	16.625 (3.986, 69.349)	<0.001	10.378 (1.399, 77)	0.022
NEC G3				
Tumor thrombus				
Yes	14.182 (2.319, 86.74)	0.004	0.603 (0.049, 7.429)	0.693
No				
AFP				
Normal	1.008 (0.993, 1.023)	0.317		
Abnormal				
CA199				
Normal	0.989 (0.938, 1.043)	0.684		
Abnormal				
CEA				
Normal	1.014 (0.687, 1.497)	0.943		
Abnormal				
CA125				
Normal	1.033 (0.969, 1.102)	0.320		
Abnormal				
NLR ≤2.056	7.429 (1.934, 28.540)	0.003	6.740 (1.298, 34.998)	0.023
NLR >2.056				
Functional tumor				
Yes	0.214 (0.026, 1.734)	0.149		
No				

*NET* neuroendocrine tumor, *NEC* neuroendocrine carcinoma, *AFP* alpha-fetoprotein; *CA199* carbohydrate antigen 199, *CEA* carcinoembryonic antigen, *CA125* carbohydrate antigen 125

### Prognostic nomogram to predict LN metastasis

**Fig. 2:**
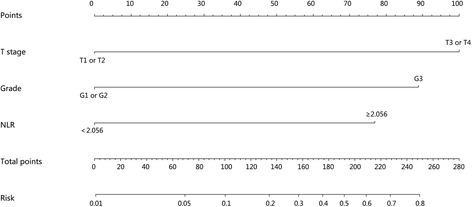
A nomogram to predict the risk of LN metastasis of PanNETs after operation. In this model, patients with high points are more likely to obtain LN metastasis

**Fig. 3:**
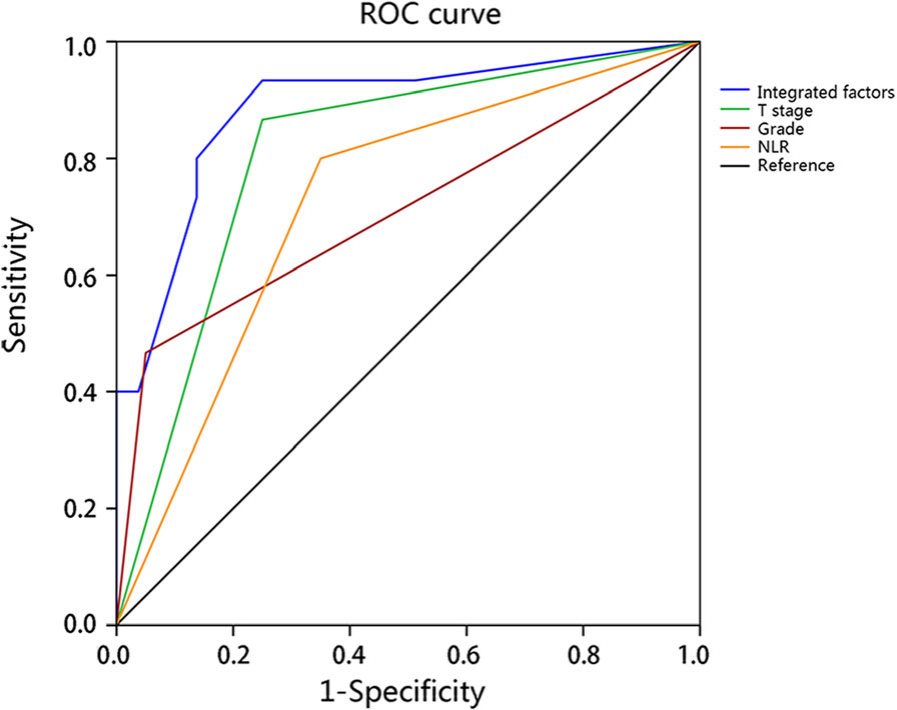
The ROC curve of the multivariate logistic regression model. The ROC curve illustrated integrated factors have an AUC of 0.885 (95% CI 0.783–0.987), which is larger than using the individual factors of NLR (0.725, 95% CI 0.591–0.859), T stage (0.808, 95% CI 0.693–0.924), and grade (0.708, 95% CI 0.541–0.876)

**Fig. 4:**
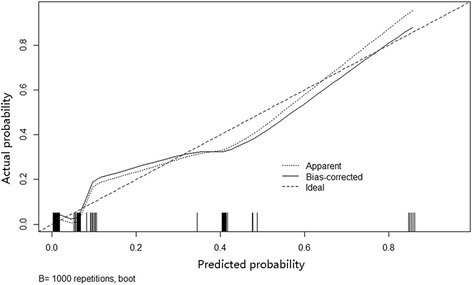
Calibration plot of nomogram. The *dashed line* indicates the ideal nomogram in which predicted and actual probabilities were perfectly identical. The *dotted line* indicates actual nomogram performance. The *solid line* presents the bootstrap corrected performance of our nomogram. The calibration plot illustrates a good predictive accuracy

Furthermore, we developed a nomogram based on the combination of these three independent factors to predict LN metastasis (Fig. 2). Specifically, ROC using the combining system demonstrated a good discrimination ability with the AUC of 0.885 (95% CI 0.783–0.987) (Fig. 3). The under-the-curve area is larger than when using the individual factors of NLR (0.725, 95% CI 0.591–0.859), T stage (0.808, 95% CI 0.693–0.924), and grade (0.708, 95% CI 0.541–0.876). In addition, calibration plot with bootstrap sampling (*n* = 1000) summarized the performance characteristics of the nomogram (Fig. 4). Our bias-corrected curve (solid line) was close to the ideal curve (dashed line). This observation indicated that the nomogram was well calibrated.

### Comparison of RFS of high NLR with low NLR after curative operation

**Fig. 5:**
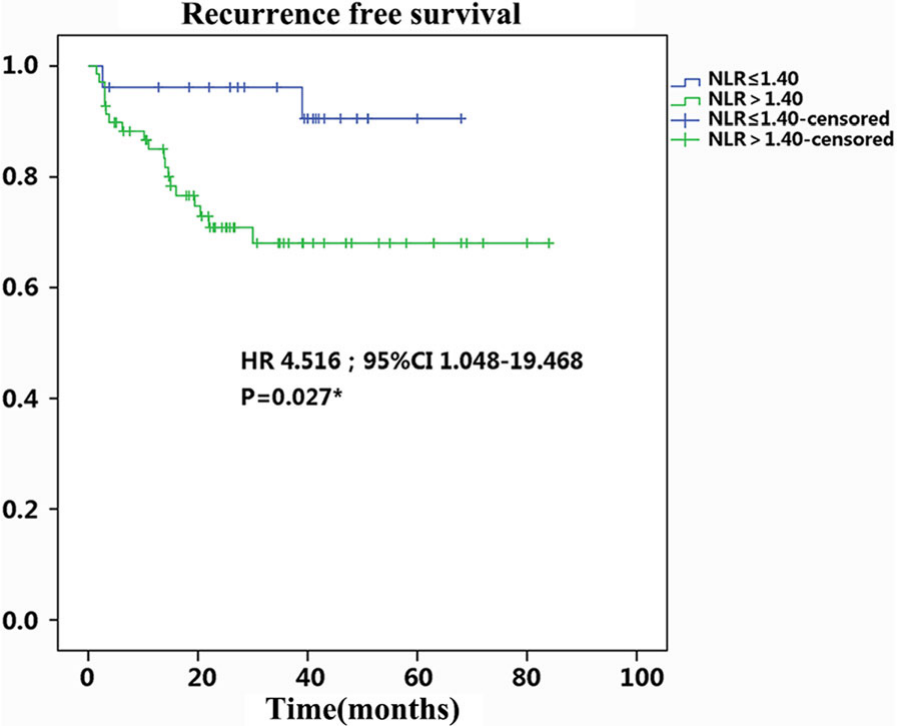
Kaplan–Meier recurrence-free survival curves for the patients with PanNETs after resection. The *survival curves* show that the patients with high NLR decreased significantly as compared with those of low NLR

The cutoff value revealed by ROC of NLR for RFS was 1.40 (Fig. 1b), and the Kaplan–Meier curve indicated that the RFS of patients with high NLR (NLR >1.40, RFS 61.1 ± 4.4 months) decreased significantly as compared with those of low NLR (NLR ≤1.40, RFS 63.8 ± 2.9 month, *p* < 0.05) (Fig. 5). The differences between the high and low NLR groups indicated NLR is a prognostic factor for resectable PanNETs.

## Discussion

**Fig. 6:**
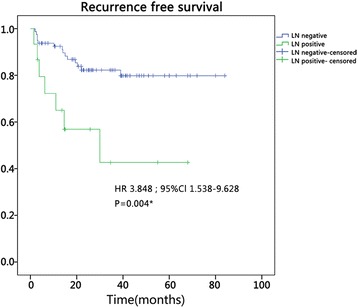
Kaplan–Meier recurrence-free survival curves for the patients with PanNETs after resection. The *survival curves* show that the patients with LN positive decreased significantly as compared with those of LN negative

With an incidence of about 0.3 cases per 100,000 in the USA, PanNET is a type of rare neoplasm accounting for a minority of overall pancreatic tumors [4]. Although WHO and ENETS published authoritative staging and grading systems, prognostication of this disease still remains unclear [10]. Our study showed patients without LN metastasis had better RFS, and this result indicates the predictive value of LN status (Fig. 6). The observation that patients with LN metastasis had increased NLR encouraged us to explore the relationship between NLR and LN metastasis (Table 2).

More evidence confirmed that the systemic inflammatory response can increase the risk of cancer [13, 28–30]. Inflammatory responses participate in different stages of tumor development, including initiation, promotion, malignant conversion, invasion, and metastasis [13]. Neutrophil is one of the dominant leukocyte subsets in peripheral blood and participates actively in promoting tumor genesis, progression, and metastasis. It can secrete various types of cytokines, including GM-CSF, TNF-α, IL-1, and IL-8 to boost tumor genesis and progression [31]. Circulating tumor-associated neutrophils can prolong tumor cell survival by suppressing the peripheral leukocyte activation [32]. Furthermore, neutrophil may facilitate tumor angiogenesis and metastasis by enhancing tumor cell adhesion to the endothelium [6, 33]. Tumor cells can be brought to the endothelium and adhere to lymphatic endothelium through interaction with neutrophils [17], and studies have noted that neutrophils can promote tumor cell adhesion to the microvascular wall by production of ROS [34]. In summary, neutrophils might serve as mediators of the LN metastasis.

Contrary to neutrophils, lymphocytes play an important role in cell-mediated immune response activation. Lymphocyte infiltration is common in NETs, as assessed by immunohistochemistry for CD3, CD4, CD8, and CD56 [35–37]. Tumor-infiltrating lymphocytes (TILs) have been shown to predict outcome in ovarian cancer, colorectal cancer, and hepatocellular carcinoma [38–40]. On the other hand, previous studies also indicated lymphopenia is associated with decreased survival [41, 42].

In combination with the effects of neutrophils and lymphocytes mentioned above, the NLR may be a potentially effective biomarker for tumor prognosis. Our data shows that NLR was an independent prognostic factor for LN metastasis and the RFS of patients with high NLR decreased significantly as compared with those of low NLR. Our conclusion concurs with other studies [17, 43]. In the meantime, our research also demonstrated that advanced T stage and grade were independent prognostic factors for LN metastasis, which is in accordance with previous studies, as well [9].

Nomogram, a new type of statistical prediction model, has been widely applied for cancer prognosis [44]. This model combines multiple relevant clinical predictors for readable and recognizable demonstrations. Because of these advantages, we established a nomogram model to evaluate the risk of LN metastasis of PanNETs by combining multiple factors of grade, T stage, and NLR. The new combined system demonstrated a good discrimination ability with an AUC of 0.885, higher than individual parameters. Moreover, the calibration plot illustrated a good predictive accuracy, which may predict a good concordance and a reliable ability to estimate the risk of LN metastasis. Our nomogram model is a simple graphical prediction tool that can be used for predicting the risk of LN metastasis of PanNETs. In this model, patients with high points are more likely to obtain LN metastasis. Therefore, this prediction system will provide more informational assistance for the surgeon to choose a more accurate and optimized surgical approach ahead. Also, patients with high NLR are more likely to be recurrent after operation, and as there is no standard adjuvant therapeutic protocol for pancreatic neuroendocrine neoplasms, NLR can be used as an assessment tool for doctors to apply adjuvant therapy.

Our study nevertheless had some limitations. Firstly, selection biases may be inevitable since our study was retrospective and hospital-based. Secondly, a multi-center study may be performed in the future to benefit the verification of our findings from this single-center analysis. Thirdly, due to the limited follow-up time, we were not able to demonstrate the significant difference between NLR and overall survival. Our future work will continue to follow up the patients’ status with prolonged observation time. Finally, the sample size is limited mostly due to the low incidence of PanNETs.

## Conclusions

In conclusion, multivariate analysis demonstrated the role of preoperative NLR as a predictor for LN metastasis. Our nomogram model highlights the important role of NLR as a convenient and reliable indicator for LN metastasis, which may help physicians to evaluate the risk of LN metastasis before operation.

## Abbreviations

AFP: Alpha-fetoprotein
AUC: Area under the curve
CA125: Carbohydrate antigen 125
CA199: Carbohydrate antigen 199
CEA: Carcinoembryonic antigen
LN: Lymph node
NEC: Neuroendocrine carcinoma
NET: Neuroendocrine tumor
NLR: Neutrophil-to-lymphocyte ratio
PanNETs: Pancreatic neuroendocrine tumors
RFS: Recurrence-free survival
SIR: Systemic inflammatory response
